# Analysis of Somatic Mutations in Cancer: Molecular Mechanisms of Activation in the ErbB Family of Receptor Tyrosine Kinases

**DOI:** 10.3390/cancers3011195

**Published:** 2011-03-10

**Authors:** Andrew J. Shih, Shannon E. Telesco, Ravi Radhakrishnan

**Affiliations:** Department of Bioengineering, University of Pennsylvania, 210 S. 33 Street, 240 Skirkanich Hall, Philadelphia, PA 19104, USA; E-Mails: shihaj@seas.upenn.edu (A.J.S.); shannone@seas.upenn.edu (S.E.T)

**Keywords:** ErbB/EGFR/HER kinase, multiscale modeling, somatic mutation, ERK/Akt activation

## Abstract

The ErbB/EGFR/HER family of kinases consists of four homologous receptor tyrosine kinases which are important regulatory elements in many cellular processes, including cell proliferation, differentiation, and migration. Somatic mutations in, or over-expression of, the ErbB family is found in many cancers and is correlated with a poor prognosis; particularly, clinically identified mutations found in non-small-cell lung cancer (NSCLC) of ErbB1 have been shown to increase its basal kinase activity and patients carrying these mutations respond remarkably to the small tyrosine kinase inhibitor gefitinib. Here, we analyze the potential effects of the currently catalogued clinically identified mutations in the ErbB family kinase domains on the molecular mechanisms of kinase activation. Recently, we identified conserved networks of hydrophilic and hydrophobic interactions characteristic to the active and inactive conformation, respectively. Here, we show that the clinically identified mutants influence the kinase activity in distinctive fashion by affecting the characteristic interaction networks.

## 1. Introduction

Lung cancer is the number one cause of death (157,000 in 2010) in America and accounts for almost as many deaths as the next three cancers—breast/prostate (40,000/32,000 in 2010), colon (51,000 in 2010) and pancreas (36,000 in 2010)—in both genders combined (American Cancer Society). About 85–90% of lung cancers are non-small cell lung cancers (NSCLCs), and present a poor prognosis in comparison to breast/prostate and colon cancers. Recent advances in cancer therapeutics targeting the EGFR/ErbB family of Receptor Tyrosine Kinases (RTKs) have shown promise in providing an alternate therapy with efficacy at least equivalent to that of chemotherapy in mutant forms of EGFR [1,2]. Here we shall focus on the intracellular kinase domain of the ErbB family receptors as a target for cancer therapy, while reviewing the overall RTK function and regulation.

### 1.1. RTK Structure and Signaling

The ErbB family kinases are a set of four homologous RTKs: EGFR/ErbB1/HER1, ErbB2/HER2, ErbB3/HER3, and ErbB4/HER4. RTKs are single pass transmembrane proteins important in intercellular signaling, by translating extracellular signals (ligands or growth factors) into activation of specific cell signaling cascades (reviewed in [3,4]). In humans, there are currently 58 known RTKs divided into 20 families. RTKs are composed of an extracellular ligand binding domain, a single transmembrane helix, a juxtamembrane domain, a cytoplasmic kinase domain and a C-terminal tail containing multiple phosphorylatable tyrosines. Activation of the extracellular domain by ligands triggers receptor dimerization and *trans*-phosphorylation of both the cytoplasmic kinase domains as well as the C-terminal tail. Dimerization and phosphorylation of the kinase domains activates the kinase domain which then phosphorylates the tyrosines in the C-terminal tail. The phosphorylated tyrosines serve as a docking site for downstream signaling molecules containing Src homology domain 2 and/or phosphotyrosine-binding domains and relay the signal into the subsequent steps of the cascade. The cell signaling pathways RTKs modulate involve crucial cellular processes such as cell proliferation, differentiation, metabolism, migration, and apoptosis.

The ErbB signaling network presents a “bow-tie” architecture, where multiple inputs and outputs are linked through a set of core processes [5]. The ErbB kinases are capable of binding a variety of ligands resulting in homo and heterodimerization, improving the flexibility and robustness in the ErbB signaling network by increasing response to extracellular signaling as well as allowing for cross-talk between ErbB kinase dimers, compensating for any reduced signaling of an individual ErbB member in a given cell type. For example, ErbB2 does not currently have a known ligand and ErbB3 is a pseudo kinase, missing key residues in the active site, greatly reducing its kinase efficacy [6]. However, ErbB2/ErbB3 heterodimers are extremely relevant in signaling and their overexpression is correlated with oncogenic transformation in breast cancers [7,8]. While response to extracellular signals occurs only through the four ErbB family kinases, which is then channeled through a conserved, relatively small collection of core biochemical interactions, the ErbB network expands again through transcription factors and positive as well as negative feedback mechanisms, eventually leading to the important cell signaling pathways of proliferation, differentiation, *etc*.

### 1.2. Temporal Regulation of RTK Signaling at the Cell Surface

Following the activation of RTKs, there are pathways which modulate the length of time the kinase is active on the cell surface, mainly regulated by receptor-mediated endocytosis and cellular phosphatase activity. Upon ligand induced activation, RTKs are internalized which removes the active RTK as well as the ligand from the cell surface (reviewed in [9-11]). The predominant pathway for endocytosis for RTKs is clathrin-mediated endocytosis, where the RTKs are rapidly endocytosed through clathrin-coated pits. One of the members of the ErbB family, ErbB4, has an alternate method of internalization by means of a proteolytic cleavage [12], which constitutes a biochemical switch and is involved in proper cardiac and neural development [13,14].

Protein Tyrosine Phosphatases (PTPs) oppose RTK activity, by removing the phosphate group on phosphotyrosines. The balance of the interplay between the RTKs and PTPs act as a major switch controlling the full activation of RTKs and thereby the cell fate decisions [15]. Prior to RTK activation, PTPs are in constant activity to reduce any residual phosphorylation from cross-talk. Given significant ligand, the RTKs inhibit/over-ride local PTP activity and have enhanced signal propagation [16]. In some cases, ligand binding also causes recruitment of PTPs that bind to target RTKs, dephosphorylates them, stabilizes the inactive form at the cell surface and inhibits further signaling [17]. The bivalent relationship between PTPs and RTKs thus forms a versatile regulatory unit in signaling.

Adding to activation and regulation of RTKs is the role of recently discovered cytoplasmic proteins cytohesins in EGFR [18] (and the proteins Dok7 in MuSK [19]). These proteins modulate RTK activity in both positive and negative fashion dependent on concentration. Increased amounts of such proteins activate the RTKs without any ligand binding events, while lacking the proteins prevents the activation of the RTKs even with a ligand binding event. It is an emerging view that cytohesins are important in the scheme of ErbB in dimerization and activation, although their specific role as an extra layer of control in the cell is unclear.

### 1.3. Regulation, Structure and Auto-inhibition of RTKs

**Figure 1.**
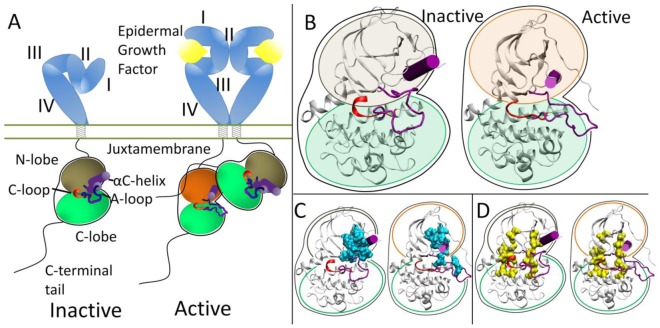
(**A**) Activation scheme for the ErbB family. The inactive kinase (brown N-lobe) is auto-inhibited through the A-loop and αC-helix (purple). Introduction of the asymmetric dimer interface rotates the αC-helix to the active state (orange N-lobe). (**B**) Enhanced view of the inactive and active kinase domains. (**C**) Hydrophobic core (cyan) in the inactive and active conformations. (**D**) C-spine (left yellow spine) and R-spine (right yellow spine) in the inactive and active conformations.

At the protein level, the extracellular domains are locked into an auto-inhibitory state preventing dimerization and are released with a ligand binding event. The specifics of how ligand binding facilitates dimerization for each RTK falls in the spectrum of “ligand mediated” dimerization, where the ligands bridge the two receptors without the receptors making direct contact, and “receptor mediated” dimerization, where the ligands make no direct contribution to the dimer interface (all mechanisms are reviewed in [20]). The ErbB family represents the extreme of “receptor mediated” dimerization [21,22]. The ErbB extracellular domain consists of four domains, with auto-inhibitory interaction between domains II and IV in a tethered conformation (Figure 1A) [23-26]. Ligands for the ErbB family are bivalent and bind to Domains I and III which cause a conformational change breaking the tethered conformation and exposing a dimerization arm in domain II allowing the dimerization arm to contact another ErbB RTK molecule.

Each RTK kinase domain is *cis*-autoinhibited in a characteristic fashion (reviewed in [20]) with activation mechanisms being unveiled as each RTK is being studied more in-depth. In RTK signaling, the intracellular kinase domain catalyzes transfer of the γ-phosphate of ATP to tyrosines on both the RTK itself and in other target substrates (reviewed in [3]). Regulation of the RTK kinase domain is thought to involve contributions from several conserved subregions: the catalytic loop (C-loop), the activation loop (A-loop), the glycine-rich nucleotide binding loop (P-loop), and the αC-helix, which together define the active site in the cleft between the β strand-rich N-lobe and the helical C-lobe. The catalytic loop residues directly participate in phosphoryl transfer. The A-loop and the αC-helix (Figure 1B) modulate the activity of the kinase domain by regulating accessibility of the active site to binding and coordinating both ATP and the substrate tyrosine. The ∼20 amino acid A-loop in ErbB kinases contains one phosphorylatable tyrosine (Y845 in EGFR, Y877 in ErbB2, Y850 in ErbB4, note: there is an alternate numbering scheme for the ErbB family, depending on whether a signaling segment is included; for example in EGFR, Y845 would be equivalent to Y869 in the alternate numbering scheme). The αC-helix and P-loop must be positioned correctly to coordinate the ATP and the substrate tyrosine for effective phosphoryl transfer.

Recent structural studies have revealed highly conserved hydrophobic “spines” within kinases that are considered important for defining their catalytic state [27,28], shown in Figure 1D. The regulatory spine (R-spine) consists of four hydrophobic side chains (M742, L753, H811, F832 in EGFR) anchored by an aspartic acid in the αF-helix (D872 in EGFR). The R-spine spans several key regulatory subdomains, and coordinates the motion of the N- and C-lobes of the kinase [27]. The catalytic spine (C-spine) involves eight hydrophobic side-chains (V702, A719, L774, V819, L820, V821, T879, L883 in EGFR) that help support and coordinate the adenine ring of ATP in the active state [28]. Similarly, in the inactive state there is a small hydrophobic ‘core’ formed between the αC-helix and the A-loop, which maintains the kinase in the inactive conformation (Figure 1C). Disruption of this hydrophobic core by single point mutations has been shown to activate EGFR [29-33].

Many of the kinase domains are inhibited through steric hindrances of a protein segment blocking off the active site and greatly reducing the efficacy of the kinase. Dimerization puts two kinase domains in close proximity to each other and although the kinase efficiency is greatly reduced, it is theorized that each kinase still has enough activity to phosphorylate its dimer partner. Phosphorylation of the protein segment prevents the protein segment from binding into the active site and allows the kinase to fully function. For the Ins [34] and FGFR [35] family of kinases, the A-loop serves as the inhibitory segment, while the juxtamembrane domain serves the same autoinhibitory role as the A-loop in MuSK [36], Flt3 [37], KIT [38] and the Eph [39] family; in the Tie2 [40] kinase, a segment of the C-terminal tail acts as the auto-inhibitor.

The ErbB family (and also Ret) has a different method of inactivation as phosphorylation of the A-loop or any other protein segment does activate the kinase; rather than a steric hindrance of the active site, there are collective auto-inhibitory interactions preventing the proper coordination between key loops in the kinase [41,42]. Activation of the kinase domain is also achieved through dimerization, though in this case, the dimer interface itself serves as the activating mechanism. In the ErbB family, the dimer interface is asymmetric similar to that of the cyclin dependent kinases and cyclin; the C-lobe of one kinase, the “activator”, contacts the N-lobe of the other kinase, the “receiver,” with the asymmetric dimer contacts causing a conformational change towards the active state through allosteric methods (Figure 1A) [43,44]. The αC-helix in the inactive ErbB kinase is rotated out, preventing key interactions from forming. Introduction of the activating asymmetric dimer interface forces the αC-helix to sample a different conformational space biasing towards the active state. Furthermore, the juxtamembrane domain in EGFR serves as latch to facilitate the asymmetric dimer interface between kinase domains [45-47].

### 1.4. Kinase Domain Mutations in Cancer and their Therapeutic Importance

Deregulation and mutation of RTKs have been correlated with cancer almost immediately after their discovery and purification in the early 1980s. The v-erbb oncogene in the avian erythroblastosis virus that was capable of inducing acute leukemia encoded a constituently active form of the homologous ErbB kinase protein [48]. With the increased study upon RTKs, the correlation between deregulation of RTKs and a variety of ailments and particularly in cancer has only grown stronger. Deregulation of RTKs in cancers can occur at several points: (1) increased ligand production through enhanced local autocrine activation; (2) specific gene translocations to produce kinase fusions with altered signaling profiles; (3) RTK overexpression at the cell surface; (4) mutation of the RTK protein to modulate activity; (5) disregulation of phosphatase and endocytosis mechanisms to increase RTK signal propagation.

**Figure 2.**
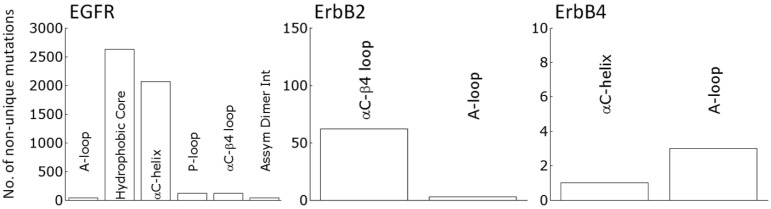
Non-unique mutations cataloged in cancer samples for the ErbB family in catalytically important sub-domains, curated from the COSMIC database.

Clinically identified activating RTK kinase domain mutations have been discovered throughout many cancers (Tables 1 and 2). The results from Tables 1 and 2 are curated from the Catalog of Somatic Mutations In Cancer (COSMIC [91]), which has a much more thorough listing for all the mutations and all cancers. The oncogenic mutations cluster near the characteristic aspects of kinase activation (Tables 1 and 2). In Kit the predominant clinically identified activating mutations are focused on the juxtamembrane domain and the A-loop, both of which alter how the juxtamembrane domain serves as a steric hindrance to the active site. Whereas in the FGFR family, the kinase mutations are around the A-loop which serves as the inactivating segment. EGFR is *cis*-inhibited through autoinhibitory interactions centered around the rotation of the αC-helix and is released by the asymmetric dimer interface and the activating mutations observed in cancers for EGFR are dominated by two mutations accounting for ∼4500 of the 5000 or so total mutations (Figure 2): a point mutation (L834R) within the hydrophobic core as well as a small in frame deletion at least involving residues 747 to 751, at the tip of the αC-helix. The ErbB2 RTK is prevented from forming heterodimers through association with HSP90 through the uniquely hydrophobic αC-β4 region [92,93], which is where the majority of the activating mutations present (Figure 2). ErbB4 is not as well studied as EGFR and ErbB2; however it has recently come under scrutiny as a potential therapeutic target. Nevertheless, there is debate as to whether ErbB4 functions as an oncogenic promoter [61] or as protection from oncogenic transformation [94].

The increased kinase activity increases the dependency of tumor upon the RTK, which becomes “oncogenically addicted [123]” and inhibition of the RTK is a viable route for cancer therapeutics. EGFR and one of its small molecule inhibitor, Gefitinib, is a canonical example of RTKs, cancer and targeted therapeutics. The initial discovery of Gefitinib in 1994 was met with much excitement as a potential cancer therapeutic since it would be a low-dose targeted oral cancer therapeutic. In two phase II clinical trials of Gefitinib for advanced non-small cell lung cancer after progression of the cancer with chemotherapy, patients overall showed symptom improvement rates around 40% and 1-year survival rates of 25–35% [124,125]. The favorable results from the phase II trials gained Gefitinib FDA approval in 2003 prior to phase III clinical trials. However, the phase III clinical trials of Gefitinib *versus* placebo as a second-line therapy did not show any statistical significance in survival in the overall population, but there was a therapeutic benefit to the sub-group of Asian non-smokers [126]. Examination of the tumors revealed sets of mutations in the EGFR tyrosine kinase domain [31-33]. The sub-set of the tumors harboring these EGFR mutations are exceptionally sensitive to inhibition through Gefitinib, so much so that Gefitinib has equal to or greater efficacy than standard chemotherapy treatments in EGFR mutation positive patients [1,2]. There are several other small molecule tyrosine kinase inhibitors (TKIs) as well as antibodies already approved by the FDA and in use in the clinical settings (Table 3).

Given the importance of the ErbB family in cancers, it is necessary to understand their activation mechanisms at the molecular level to help design higher specificity therapeutics. This is especially important recently as, after sustained use of TKIs, the cancers tend to adapt through resistance mutants. In EGFR, the main mutation seen after extended treatment with Gefitinib is the T766M mutation [127]; the T766M resistance mutation was correctly predicted in EGFR through homology with resistance mutations seen in BCR-ABL, which was verified *in vitro* [128] and discovered several years later in patients [129-131]. Computational methodologies offer a powerful, quantitative, and complimentary alternative for the study of intracellular kinase domains which, if utilized correctly, can predict resistance mutations [132]. Here we review our recent results investigating the hydrophilic and hydrophobic networks using molecular dynamics simulation techniques as well as signal network models to help differentiate the conformational states across the ErbB family and prove the importance of understanding somatic mutations in the ErbB family.

## 2. Results and Discussion

### 2.1. The Inactive and Active Conformations Have Distinct, Characteristic Protein Motions

We hypothesized that the atomic fluctuations in the inactive and active forms of the ErbB family kinases would differ dramatically, as conformational rearrangement of the kinase domain is expected to correlate with significant changes in the dynamical behavior of the protein. Root-mean-squared deviation (RMSD) analysis of the overall kinase motion as well as individual sub-domain motions in our molecular dynamics (MD) simulations (see Section 4) showed all of the kinase systems were stable over the course of the simulation. Principal component analysis (PCA) of the kinase systems was performed for the C*_α_* atoms of the entire kinase domain, but the majority of the fluctuations for the first few eigenmodes focused either on the free floating N-terminal and C-terminal tails or an active site consisting of sub-domains critical for catalysis, including the A-, C-, and P-loops and the *α*C helix [133]. We observed that the first three eigenmodes account for the majority of the atomic fluctuations in each trajectory, and the top 10 eigenmodes account for 75–85% of the total variation in each system.

**Figure 3.**
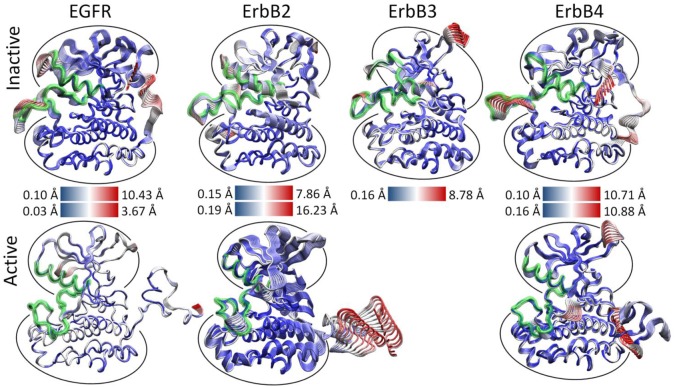
Projection of the first principal component of the MD trajectory for the ErbB monomer kinase systems. The simulations are fully atomistic, solvated and charge neutral; water and ions are not shown here. The structures are color-coded according to the RMSD, where red regions indicate large-amplitude fluctuations and blue regions indicate small-amplitude fluctuations. The active ErbB systems show a highly coordinated active site with no majority of the motions focused only on the freely movable *N*- and *C*-terminal tails. The inactive canonical ErbB (EGFR, ErbB2, ErbB4) systems have large local motions concentrated on the A-loop and αC-helix in addition to the tail motions. Despite the ErbB3 kinase in an inactive-like conformation, the ErbB3 kinase has low-amplitude fluctuations and a more coordinated active site similar to the active systems.

As evident, there is a stark difference between the inactive and active conformations of the ErbB family. The active conformations have only low-level fluctuations within the kinase with the majority focused on the freely moving N-terminal and C-terminal tails (Figure 3). The inactive not only has motion in the tails, but also has significant motion in the active site: A-loop, C-loop, P-loop and αC-helix. A focused look at the PCA of the active site of the ErbB kinases revealed that motions occurring within the active sites of the inactive and active kinase monomers differ significantly, particularly in the A-loop [133-135]. The inactive ErbB kinase monomers (except ErbB3) exhibit large-amplitude motion in both the αC-helix and the A-loop, with smaller fluctuations in the P-loop and C-loop. We attribute the larger amplitudes to a more flexible protein segment; this is consistent with the observation that part of the activation loop is unresolved in almost all of the crystal structures of ErbB kinases to date. In contrast, the active ErbB monomers demonstrate a uniform level of motion across all four subdomains of the active site with only low-amplitude fluctuations and show no significant local deformations. This implies that the motions are more tightly coordinated across the active site in the active conformation than in the inactive conformation. The inactive ErbB3 kinase monomer also exhibits coordination of its active site loops without much local deformation, more similar to the active motion than the inactive motion, though its protein conformation is distinctly not in the active conformation.

Thus, for the three canonical kinase members of the ErbB family, which have a high-degree of sequence similarity, not only are the principal motions conserved across the systems, but the characteristic differences between the inactive and active kinase conformations are maintained in character. This finding suggests large (and possibly similar) differences in the internal network of interactions between the two activity states of each kinase.

### 2.2. Hydrophilic Interaction Networks Between Catalytically Important Sub-domains Help Coordinate Motions in the Active Kinases and Sequester Key Residues in the Inactive States

We find that several interactions are conserved across the canonical kinase members of the ErbB family in the active state (EGFR numbering is used in this discussion: see Table 4). Two salt bridges: E734-K851 and E738-K721, three H-bonds: L834-R812, K836-V810, and L838-R808, as well as the interaction D813-R817 which is a salt bridge in EGFR and ErbB4, but an H-bond in ErbB2. The E738-K721 salt bridge is highly conserved across all active kinases and helps coordinate the α and β phosphates of ATP bound in the active site. The E734-K851 salt bridge connects the A-loop and the αC-helix, coordinating the movements of these two sub-domains and dampening larger fluctuations. Similarly, three conserved H-bonds link the A-loop and the C-loop, coupling the motions of these two loops. These can be regarded as “fastening” H-bonds that maintain the N-terminal side of the A-loop open in its active state—the alternative (in the inactive state) being steric hindrance to the binding of ATP and peptide substrates.

In contrast with the case for the active configurations, few intramolecular interactions in the inactive kinase conformation are conserved across the ErbB family (Table 4). The inactive kinases do share an autoinhibitory pattern in which key residues required for kinase activation are ‘sequestered’, mainly breaking up the E738-K721 salt bridge. Similar to the situation described for Lck [136,137], E738 of EGFR is salt bridged to K836 in a manner that sequesters this glutamate and prevents it from forming the highly conserved E738-K721 salt bridge [134] required for full activation. In ErbB2, ErbB3 and ErbB4, the other half of the highly conserved salt bridge is sequestered; namely, the homologous K721 interacts with the D831 side chain, and this interaction in turn prevents K721 from forming the coordinating salt bridge.

The αC-β4 loop in the ErbB2 kinase is a hydrophobically unique region compared to the other ErbB family kinases and is important in association with the molecular chaperone HSP90 to prevent heterodimerization with the other ErbB members [92,138]. The ErbB2 kinase domain shares 83% sequence identity with EGFR; in the *α*C-*β*4 loop, however, five of the eight residues in ErbB2 differ from those in EGFR. In particular, the polar residues in the *α*C-*β*4 loop of EGFR are replaced by nonpolar residues in ErbB2, which form a hydrophobic patch that interacts with another segment of hydrophobic residues positioned in the A-loop. The hydrophobic patch in ErbB2 includes the following residues: V773, M774, G776, V777, G778, and V782 in the *α*C-*β*4 loop, and I861, T862, F864, L866, and L869 in the A-loop.

In ErbB2 only the S783-I861 interaction is shared by the inactive and active conformations and links the *α*C-*β*4 loop to the A-loop [134]. EGFR and ErbB4, by contrast, reveal a stronger hydrogen bonding network in the *α*C-*β*4 loop. The active EGFR and ErbB4 systems contain 10 and eight hydrogen bonds, respectively. The active ErbB2 system lacks these hydrogen bonds because hydrophobic interactions, rather than hydrophilic contacts, predominate in the *α*C-*β*4 region (see Section 2.4 for a hydrophobic analysis).

The interaction-networks highlight key conserved residues and interactions that are characteristic of each conformational state. Overall, in the active conformation, the six conserved interactions tightly couple the N-lobe to the αC-helix, the αC-helix to the A-loop, and the A-loop to the C-loop. The nature of this interaction network suggests that interactions among the nearest key subdomains in the ErbB family help to maintain the proximity of important catalytic residues, and position them so they can contribute directly to kinase activity. The conserved interaction network in the inactive conformations of the ErbB family kinases is less extensive (in EGFR, E738-K836 and D831-K721), but appears to serve a crucial role in sequestering key catalytic residues and thus preventing activity. The ErbB3 kinase hydrophilic interaction patterns fall in-line with those of the inactive kinase while presenting a more coordinated active site. Therefore we hypothesize that other interactions, mainly hydrophobic (see Section 2.4) help distinguish the canonical ErbB kinases from ErbB3.

### 2.3. Dimerization Disrupts the Inactivating Interaction Networks

We also analyzed fluctuations in the active and inactive EGFR [135], inactive ErbB2 [134] and inactive ErbB4 [134,135] kinase within the context of the asymmetric dimer described by Zhang *et al.* [42]. The fluctuations recorded for active EGFR in this context are very similar to those seen in the active EGFR monomer, with the conserved interactions described above being mostly preserved. In contrast, in the inactive dimers there is substantial motion of the αC-helix that is much greater than seen in the inactive monomer system. Even in the short timescale of the dimer trajectories, we observe a rearrangement of the αC-helix position towards the active conformation. Consistent with the allosteric activation mechanism proposed by Zhang *et al.* [42], several interactions in the inactivating interaction network surrounding the A-loop and the αC-helix are indeed disrupted in the inactive EGFR dimer trajectory, including Y740-S744, L834-D813, H846-R865, and K851-R812 interactions. Some interactions (e.g., E738-K836) are still present, although the population statistics indicate that their survival percentage (fraction present in the trajectory) has decreased significantly. The ErbB2 and ErbB4 inactive dimers demonstrate a similar loss of interactions surrounding the αC-helix and the A-loop. For ErbB4, a list of interactions disrupted upon dimerization includes: E739-R841, D742-R841, E743-R817, G838-R817, G855-E730, and K856-E844. Similar to the E738-K836 salt bridge in EGFR, the E743-R841 salt bridge in ErbB4 shows a marked decrease in survival time from >90% in the monomer trajectory to ∼70% in the dimer trajectory. Overall the introduction of the asymmetric dimer interface to the inactive ErbB kinases results in a significant weakening of the interactions in the inactivating interaction-network (discussed above) which sequesters key side-chains in the inactive state.

### 2.4. Hydrophobic Interaction Networks Help Differentiate between Active and Inactive Conformations

**Figure 4.**
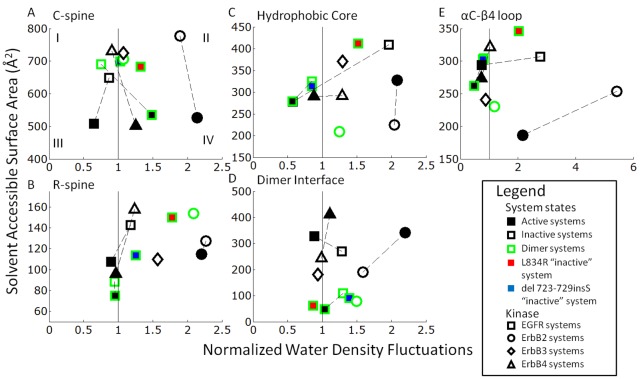
Hydrophobic plots of the solvent accessible surface area (SASA) *versus* normalized water density fluctuations in the key hydrophobic regions of the ErbB kinases: (**A**) C-spine; (**B**) R-spine; (**C**) Hydrophobic Core; (**D**) Dimer Interface; and (**E**) αC-β4 loop. The edge colors represent monomer (black) and dimer (green) systems. The shape internal color represents active (black) and inactive (white) for the wildtype systems. The mutant systems of EGFR L834R in the inactive conformation are red while the EGFR del 728-729insS mutant system is blue. The shape itself represents a different member of the ErbB family EGFR (square), ErbB2 (circle) and ErbB4 (triangle). The quadrants represent different hydrophobic interaction regions (I) hydrophilically favorable region, (II) perturbation-sensitive region and (IV) hydrophobically favorable region. It is not clear if quadrant III represents a favorable or unfavorable region as it represents a buried hydrophilic surface.

Hydrophobic interactions appear to provide context-specific contributions to stability of the active and inactive conformations of ErbB kinases. To investigate the effect of hydrophobic interactions on the ErbB kinase conformations, we analyzed the hydrophobicity as well as the solvent accessible surface area (SASA) of relevant hydrophobic sub-regions, namely, the C-spine, R-spine, hydrophobic core, and the αC-β4 region [133,135]. The four regions have a high percentage of hydrophobic side chains; however, some minor differences between members of the ErbB family exist, particularly in the αC-β4 region. The hydrophobicity of the sub-region is a non-additive quantity, dependent not only upon the primary structure but also on the surrounding environment. Garde *et al.* [139,140] have recently proposed an approach for quantifying the hydrophobicity of heterogeneous surfaces using water density fluctuations, according to which increased normalized water density fluctuations are used as a signature of a more hydrophobic surface. We have normalized the water density fluctuations so that 1 is indicative of a neutral region and plotted them against the SASA. By splitting the graph into four quadrants (Figure 4A) we can see quadrant I represents a hydrophilically favorable region with low hydrophobicity and high SASA, while quadrant IV represents a hydrophobically favorable region with high hydrophobicity and low SASA. Quadrant II represents a fragile or perturbation sensitive region with high hydrophobicity but also with a high SASA.

The active conformations in the C-spine show a clear delineation between the active and inactive conformations regardless of dimer or mutational state (Figure 4A). The active conformations minimize the SASA (mean of 500 Å^2^) in comparison to the inactive (mean value of 700 Å^2^). In addition, in the transition between the inactive to active states, the active conformations increase in hydrophobicity and settle into the hydrophobically favorable region, implying the active conformations have a better formed C-spine, correlating with the observation that the C-spine helps in coordinating the loops in the active conformation. The SASA of the ErbB3 C-spine falls within range of the inactive EGFR and ErbB4 systems, reflecting that, there is no corresponding ‘fully-active’ state for ErbB3 as for the other ErbB kinases. This inability to ‘fully’ activate can be attributed to the lack of the crucial hydrogen bonding network identified earlier, which is required to stabilize the active-like kinase conformation. The hydrophobicity plots of the R-spine show a similar difference between the active and inactive conformations but not as drastically as the C-spine (Figure 4B). For each system the active systems expose less surface area than the inactive systems, but only for each system locally (dotted lines on Figure 4). The SASA of the ErbB3 R-spine deviates from the values for the inactive EGFR and ErbB4 systems, and instead demonstrates low SASA (high hydrophobicity). This result can be rationalized by the increased hydrophobicity of the R-spine, which includes segments of the truncated αC helix in ErbB3.

With respect to the hydrophobicity plots of the hydrophobic core, the monomer EGFR and ErbB4 inactive systems are situated within the perturbation sensitive region with their respective active conformations lying outside of the perturbation sensitive region (Figure 4C). For ErbB2 the reverse is true, which is consistent with the sensitivity of the inactive conformation to hydrophobic perturbation, especially for EGFR and ErbB4 but not for ErbB2. Therefore single point mutations would be expected to disrupt local conformations contributing to hydrophobicity. Notably, mutations of hydrophobic residues in the hydrophobic core are reported for EGFR and ErbB4 in clinical studies [31-33], whereas, for ErbB2, such mutations are found surrounding the αC-β4 region [57,58]. By contrast, an analogous mutation in ErbB3 abolishes ATP-binding and phosphorylation activity [141], indicating that hydrophobic interactions in the core promote ErbB3 activity, rather than maintain an autoinhibited state as they do in EGFR and ErbB4.

So far all the hydrophobic plots have shown preferential hydrophobic interactions in the active systems *versus* the inactive systems. However, examination of the asymmetric dimer interface reveals a clear delineation in hydrophobicity in preference for the inactive systems as well as dimerization reducing this hydrophobic benefit implying an activating stimulus (Figure 4D). The asymmetric dimer interface consists largely of hydrophobic side-chains in the N-lobe of the receiver kinase (L680, I682, L736, L758, and V762) and the C-lobe of the activator kinase (I917, Y920, M921, V924, and M928) [42]. The active monomer systems trend to the perturbation sensitive quadrant, while the inactive monomer systems show a reduced SASA in comparison to the active systems, removing them from the perturbation sensitive quadrant. This decrease for the inactive monomers may imply a preference for the inactive state in the monomer context. Notably, the dimeric systems record much lower SASA, pushing all the dimer systems into the hydrophobically favorable quadrant, implying that hydrophobic interactions provide a dominant driving force for dimerization; interestingly, for the EGFR dimer, the inactive state is very similar to the active and hence the preference for the inactive conformation is not implied in the context of the dimer. Thus, dimerization also provides a stimulus for activation.

Interestingly, all ErbB2 monomer systems present a very high hydrophobicity (black bordered circles on Figure 4) across all of the hydrophobic subregions, while the ErbB2 dimer has a reduced hydrophobicity in comparison to the monomers (green circles on Figure 4). This is consistent with the notion that hydrophobicity is particularly important in the context of ErbB2, owing to its hydrophobic interaction with HSP90, particularly the αC-β4 region which is an unstructured span between the αC-helix and the β4 sheet in RTKs. From a sequence perspective, only in ErbB2 is the αC-β4 region predominantly hydrophobic and exceptionally so. Both the inactive and active conformations of the ErbB2 monomer systems reflect the trend by singling out the ErbB2 monomer systems as particularly hydrophobic in the αC-β4 region (Figure 4E). The mean SASA for the αC-β4 region in the ErbB2 systems is also consistently lower than in other members of the ErbB family. As discussed in [134], this unique feature of ErbB2 is thought to be responsible for its preferential association with the molecular chaperone Hsp90.

Thus, the analyses for the spine regions and the hydrophobic core collectively lead to the remarkable prediction that while the αC-β4 region is perturbation sensitive for the inactive conformation of ErbB2, the hydrophobic core has the same effect for EGFR and ErbB4. Indeed, this correlates well with clinical studies, where activating point mutations in the hydrophobic core have been found in EGFR and ErbB4 but those in the αC-β4 region are found in ErbB2, suggesting that the hydrophobic analysis enables the context-specific identification of fragile sub-regions.

### 2.5. A-Loop Phosphorylation in EGFR and ErbB2 Reveals Extra Domain Coupling

The role of A-loop phosphorylation in the ErbB family kinases is controversial. Phosphorylation of EGFR on Y845 (Y877 in ErbB2 and Y850 in ErbB4) has been observed experimentally, though does not seem to be required for catalytic activity, as a Y845F mutation is fully active [142]. In contrast, multiple studies have reported the importance of Y877 phosphorylation for kinase activity [143,144], though the precise mechanism of phosphorylation is not clear. Hence it is possible that phosphorylation of Y877 potentiates ErbB2 kinase activity.

To investigate the effects of A-loop phosphorylation in the ErbB family, we simulated EGFR [135] and ErbB2 Y845-phosphorylated and Y877-phosphorylated systems [134], respectively, both in the inactive and active conformation. In the active A-loop phosphorylated systems, we discovered that they mostly preserved the same set of a network of hydrophilic interactions seen in the unphosphorylated systems, but also gain several more fastening interactions that maintain the *C*-terminal side of the A-loop in the open conformation: the ErbB2 systems gain Y877-F899, A879-R897 as seen in [134]; the EGFR phosphorylated systems have the homologous fastening interactions in Y845-Y867 as well as A879-R865. All of the phosphorylated systems (active and inactive systems) share a common interaction in Y845-R812, which strengthens the coordination between the A-loop and C-loop. The inactive systems do see a shift in interaction patterns, but no subsequent weakening of the sequestration interactions. Therefore we hypothesize that while phosphorylation incrementally strengthens the coordination of the active site, it is not a strong enough stimulus to cause activation.

To quantify the effect of A-loop phosphorylation on ErbB2 activity, we computed the Helmholtz free energy difference between the Y877-unphosphorylated and Y877-phosphorylated states in the NVT ensemble using the FEP method [134]. Four different simulations were performed for comparison, including the phosphorylation transformation of Y877 to pY877 in the unphosphorylated structures (inactive and active) as well as the respective pY877 to Y877 de-phosphorylation transformation in the phosphorylated systems. The transformation of Y877 to phosphorylated Y877 produced a free energy change ΔΔ*F* value of −1.1 ± 1.4 kcal/mol [134]. The free energy values (ΔΔ*F* values) suggest that phosphorylation of Y877 provides a small increase in stability of the active conformation relative to the inactive state, although it is insufficient to significantly lower the kinase activation barrier. The free energy studies support our hypothesis that phosphorylation does not cause activation, but does marginally increase coupling of key loops in the active site. Consequently, we suspect molecular stimuli in addition to A-loop phosphorylation are likely required for the complete conformational activation of the ErbB2 kinase.

### 2.6. Effects of Clinically Identified Somatic Mutations on the Interaction Networks

The inactive and active conformations are differentiated by the balance between the hydrophobic and hydrophilic interaction networks [135], with perturbation by A-loop phosphorylation not greatly altering these networks. Here we examine what happens to these networks with the perturbation by clinically identified activating mutations [135]. The predominant mutations in the clinically identified EGFR activating mutations, a small in-frame deletion at the start of the αC-helix (we used del 723–729 ins S), alter both the hydrophilic and hydrophobic interaction networks. The L834R mutation increases the coupling of the A-loop and C-loop (through the G833-H811 H-bond) and the A-loop to the C-lobe (through the R834-R865 interaction) not seen in other systems. The hydrophobic interaction networks are disrupted because of the switch of the hydrophilic Leu to a hydrophilic Arg, particularly in the R-spine and the hydrophobic core, shifting them into the perturbation sensitive region, indicating a stimulus for a conformational shift (Figure 4B and 4C). The deletion mutant directly alters the conformation of the αC-helix by shortening the connecting subdomain, thereby shifting it towards the active conformation. However because it is missing residues in the αC-helix, the dynamics and the potential extension of the αC-helix are significantly inhibited. In studies by Choi *et al.* [30], the deletion mutant had increased basal kinase activity, but under EGF stimulation it had less overall kinase activity compared to wildtype. In the monomer state, the deletion mutant causes a shift towards active, disrupting key interactions and potentially increasing activity; once the asymmetric dimer forms, the active conformation is destabilized, through the deformation of the αC-helix interfering with the dimer interface and lack of a completely formed helix.

Other EGFR mutations in NSCLC [30,31,49] (E685S, G695S, S744I and L837Q) plus a set of mutations in ErbB4 found in melanoma [61] (E836K, E872K and G936R) also alter the hydrophilic and hydrophobic interaction networks and allow us to propose a set of mechanisms for their increased basal kinase activity. For the L837Q mutation, the replacement of the hydrophobic Leu with the hydrophilic Gln would disrupt the hydrophobic core between the A-loop and the αC-helix, with the Q837 side-chain rotating away and inducing a local steric effect; similar to L834R the Q837 residue may cause increased coupling between the active site subdomains and also disrupt the interaction of K836 with E738. The S744I mutation in NSCLC is situated at the base of the αC-helix and alteration of hydrophobicity likely causes a transition of the αC-helix into its active position. Two of the mutations E685G and G695S are in the N-lobe, near the asymmetric dimer interface, and are likely to alter kinase activity by either increasing the dimerization affinity or by reconfiguring the EGFR RTK monomer by partially mimicking the formation of the asymmetric dimer interface. The G936R mutation in ErbB4 is located in the C-lobe, but is close to the asymmetric dimer interface and potentially increases the dimerization affinity between kinase domains. The E836K and E872K ErbB4 mutations are situated around the homologous small hydrophobic core and are poised to disrupt the hydrophobic interaction by the change from a negatively charged side chain to a positively charged chain, which could provide an activating stimulus.

Clinically identified mutations in ErbB2 are predominantly found in αC-β4 region (see Figure 2) [56,145], shown here to be uniquely hydrophobic in the ErbB family. The most common mutant is an in-frame insertion at residue G776 (G776^YVMA^). We hypothesize these mutations operate by weakening the hydrophobic interactions surrounding the *α*C-*β*4 loop and forming a hydrophilic interaction network similar to those we have observed in EGFR and ErbB4. Alteration of the hydrophobicity of the αC-β4 region would disrupt the binding to HSP90 [93] and allow ErbB2 to heterodimerize freely with other ErbB members [92] and thus, activity of ErbB2.

Another set of mutations in ErbB4 found in NSCLC are not activating mutations, but inactivate the kinase domain: G802dup and D861Y [94]. D861 is the start of the highly conserved DFG motif in RTKs (considered the coordinating aspartate), with mutation poised to disrupt proper ATP placement and reduce activity. The G802dup is spatially located near the P-loop and similarly affects ATP binding affinity. In ErbB4, two of the point mutations are located near the active site: E836 is located next to the C-loop, whereas E872 is situated in the A-loop. Considering their proximity to the active site, the mutations are also poised to alter substrate binding and phosphorylation; these mechanisms are discussed in a recent computational study in the context of EGFR [146].

### 2.7. Clinical Implications of Oncogenic EGFR Mutations from a Multiscale Model of ErbB Receptor Signaling

**Figure 5.**
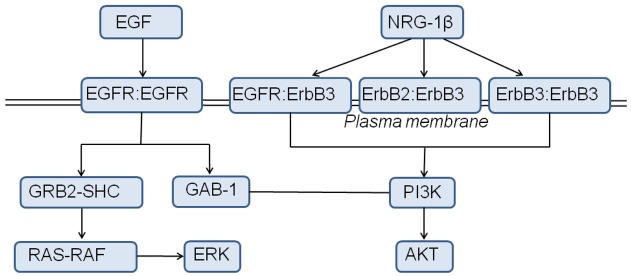
Schematic diagram of our EGFR signaling model as well as our ErbB3 signaling model. The differential ligands (EGF and NRG-1β) cause differential heterodimerization between members of the ErbB family triggering their respective cell signaling pathways.

For cellular homeostasis, pro-survival signals are balanced by pro-apoptotic signals, with both being triggered and balanced by a variety of interacting intracellular pathways. Using a simplified model (Figure 5) for the effect of AKT activation on cell response, we showed that preferential AKT activation is conducive for the cell to rely on and be addicted to [123,147]) for generation of pro-survival signals [148]. Our simplified model illustrates a mechanism by which inhibition of the dominant source of pro-survival signals shifts the cellular state to one devoid of pro-survival signals, representing a perturbation sensitive point in the cellular signaling network which can account for a remarkable inhibitor sensitivity [149].

We hypothesized the mechanisms that lead to inhibitor hypersensitivity (Gefitinib for EGFR) attack these perturbation sensitive points of network hypersensitivity and fragility. Since preferential AKT activation is a hall mark of the hyper-sensitive mutants as well as the efficacy of the inhibitors, we determined, through a global sensitivity analysis [149,150], the combinations of model parameter perturbations that drive enhanced production of pAKT and pERK. The top components that produce maximum sensitivity in terms of changes to the pAKT and pERK levels were PI3K, Ras, Gab-1, MEK, Raf which have all been observed in several human cancers [151-155]. Moreover, it has been established in screened breast and colorectal cancer patients that the GAB-1, MEK, and Ras mutations are non-random and likely arise from selective evolutionary pressures that give the cancer cells a survival advantage [155].

With reference to the clinically identified EGFR mutants found in non-small cell lung cancer patients, mainly L834R and del L723-P729 ins S, we found the mutations had altered affinities for phosphorylation of specific tyrosines in the C-terminal tail: Y1068 and Y1173. The preferential binding characteristics of different cytosolic substrates to different phospho-tyrosine locations of the ErbB family kinases has been reported [156]. Thus, differences in the phosphorylation kinetics associated the different tyrosine sites of the cytoplasmic C-terminal tail of the EGFR kinase can induce differential patterns of downstream signaling leading to differences in the activation of cell signaling networks. The effect of altered affinities of the Y1068 and Y1173 sites to the catalytic domain of the EGFR is that the L834R under normal EGFR expression sees a ∼5-fold decrease in ERK activation and a smaller ∼15% decrease in AKT activation. The del L723-P729 ins S mutant however, shows sustained ERK as well as AKT activation relative to wildtype. For EGFR over-expressed cells, both ERK and AKT activation characteristics show relative insensitivity to EGFR as a result of signal saturation. Furthermore, the mutants can continue to signal even in the absence of the growth factor. In addition, the mutant signaling can be different due to changes in the ATP affinity. However, both these factors do not introduce any differential characteristics (in terms of preferring Y1068 to Y1173) and cause a differential in overall activation levels of ERK and AKT.

The perturbation of the phosphotyrosine kinetics of Y1068 and Y1173 through mutations (L834R and del L723-P729 ins S) as well as trafficking (which is not explicitly considered in our model) could be directly responsible for the differential signaling leading to preferential AKT activation [148]. The restoration of signaling has also been reported through a double mutation of L834R/T766M [128,157]. This double mutant increases receptor phosphorylation (Y1068 and Y1173) kinetics 100-fold [158] while simultaneously decreasing inhibitor affinity [157]. Another drug resistance mechanism related to Y1068 kinetics that circumvents Y1068 has been identified. In the presence of ErbB3, a branch of signaling analogous to that through Y1068 becomes available through ErbB hetero-dimerization, directly resulting in PI3K recruitment on ErbB3 and subsequent AKT activation, which is discussed below.

### 2.8. Systems Model of ErbB Signaling Defines a Mechanism for ErbB3-mediated TKI Resistance

Previous experimental studies have demonstrated that ErbB3 is a key mediator of resistance to various tyrosine kinase inhibitors (TKIs) currently in use [159-163]. Some postulated mechanisms include leaky ErbB2-catalyzed phosphorylation of ErbB3, e.g., incomplete inhibition of ErbB2 catalytic activity by the TKI [159,162,164]. Indeed, previous experimental studies have demonstrated that leaky ErbB2 phosphorylation of ErbB3 in TKI-bound ErbB2/3 heterodimers is amplified by additional resistance mechanisms, such as inhibition of cellular phosphatases by TKI-mediated production of reactive oxygen species (ROS), and increased expression of ErbB3 at the plasma membrane [159,165,166]. However, Shi *et al.* [141] have recently shown the assumed inactive ErbB3 pseudokinase is, in fact, a weakly active kinase with ∼1000 fold weaker phosphorylation than the canonical kinase members of the ErbB family [141]. Therefore, though the ErbB2 kinase is a viable route of resistance, here we consider ErbB3 catalytic activity in the ErbB signaling network as a potential TKI resistance mechanism [133].

To translate our observations of the weak, yet robust, activity of the ErbB3 kinase into a physiologically relevant context and investigate the implications of ErbB3 activation for ErbB signaling dynamics, we constructed a systems-level model for ErbB3 based on that of Schoeberl *et al.* [167], with added ErbB3 phosphorylation rate constants derived from experiments [141] (Figure 5). We simulated stimulation of the ErbB signaling network through NRG-1β, which signals through the ErbB2/ErbB3/ErbB4 kinases (though ErbB4 kinase is omitted from the model since ErbB4 signaling is weak or absent in many cancer cell lines).

A parameter sensitivity analysis identified the key proteins that direct signaling in our model of the ErbB network with respect to AKT activation via stimulation of NRG-1β. ErbB3 and NRG-1β represent the most sensitive species in the signaling network, followed by ErbB2 concentration. EGFR is not a strong determinant of the extent of AKT phosphorylation, as expected from the weak ability of NRG-1β to elicit EGFR dimers. PTEN (the PIP_3_ phosphatase) and the ErbB phosphatase exhibited a negative sensitivity in the analysis, as these phosphatases negatively regulate the signaling network through dephosphorylation of key molecular species.

Upon incorporation of the TKI lapatinib, which inhibits EGFR and ErbB2 catalytic activity, into our model of ErbB3, the ErbB signaling network relies more heavily upon ErbB3 activity for AKT induction. With completely inhibited EGFR and ErbB2 signaling via introduction of lapatinib into the model, ErbB3 and AKT signals still persist at maximal inhibition by lapatinib. The results of pAKT sensitivity analysis of the lapatinib-treated model to those of the inhibitor-free model show that sensitivity to ErbB3 and NRG-1β increases, whereas sensitivity to EGFR and ErbB2 decreases, as lapatinib sequesters EGFR and ErbB2 molecules. The negative normalized sensitivity to the ErbB phosphatase also increases, as the pool of ErbB dimers has diminished due to sequestration of EGFR and ErbB2 by lapatinib. Thus a single alteration to the signaling model (in this case, addition of lapatinib) significantly redefines the most perturbation-sensitive nodes in the network.

Although the pAKT signal induced by ErbB3 phosphorylation in our *in silico* lapatinib-treated cell is relatively weak, in an actual physiological context, a tumor cell may employ several resistance mechanisms at once [159,162,168,169]. One of the sensitive nodes of NRG-1β-driven ErbB signaling is ErbB phosphatase levels, and simulations showed decreased activity of phosphatases resulted in an amplification of the level of AKT signaling induced by ErbB3. It has been demonstrated that in certain cases of TKI resistance, the tumor cell responds to the reduction in pAKT levels by upregulating vesicular transport of ErbB3 from the cytoplasm to the plasma membrane [159,166]. Our results show for a 2-fold increase in surface ErbB3 level, AKT signaling is similarly amplified. For 25 nM NRG-1β, the pAKT signal is restored to nearly 60% of its no-inhibitor control level, and pAKT levels are nearly 100% regained for 100 nM NRG-1β, effectively recreating drug resistance *in silico*.

Our data parallels the experimental studies performed by Sergina and colleagues [159], which describe ErbB3-mediated resistance and pAKT signaling in various TKI-treated tumor cell lines as well as *in vivo*. Thus our model demonstrates that even a weak level of ErbB3 signaling, as suggested by our previous results [141], are physiologically relevant in the context of an ErbB-driven tumor cell, and illustrates several routes through which ErbB3 signaling may be compounded by other previously postulated resistance mechanisms to generate TKI resistance.

## 3. Conclusions

Understanding the activation mechanisms of the ErbB kinases is important considering the crucial cell signaling pathways they control. Mutations of the ErbB kinases, and particularly EGFR, have been revealed in mutational screens of multiple cancers (see Tables 1 and 2). The regulation of the ErbB kinases is maintained through a flexible multiple-point network with multiple redundancies to ensure signal propagation. Here we reviewed a hierarchical multiscale model of the ErbB family kinases to investigate their activation mechanisms and the subsequent effects of kinase perturbation on their cell signaling network.

At the molecular level, the active *versus* the inactive conformations of the canonical ErbB kinases (EGFR, ErbB2, ErbB4) have a distinct fluctuation pattern, where the active conformations demonstrate low levels of fluctuations and coordination between the subdomains dictating the active site (A-loop, P-loop, αC-helix and C-loop), while the inactive conformations exhibit high-levels of fluctuations with little coordination in the active site. The similarity is carried through to the hydrophilic and hydrophobic interaction networks. The interaction networks also highlight perturbation sensitive points in EGFR which correlate well with clinically identified activating mutations in NSCLC. Simulation of the clinically identified EGFR mutations in the ErbB system model reveals enhanced dependence on the EGFR for AKT signaling; thus they are perturbation sensitive points at the cell signaling level and are sensitive to inhibition by TKIs.

The ErbB3 kinase presents a different case from the canonical ErbB kinase members, as it is missing key catalytic residues. Even though the ErbB3 kinase is distinctly in the inactive conformation with a correlated inactive hydrophilic interaction network, it exhibits a coordinated active site which seems to stem from increased hydrophobic interactions, in line with our recent studies [141] showing ErbB3 does have kinase activity, though ∼1000 fold less than the canonical ErbB kinases. At the cellular level, a weak level of ErbB3 activity may be sufficient to induce drug resistance in the context of an ErbB signaling-dependent tumor cell.

The multiscale model we present is useful in examining atomic level protein characteristics and carrying them over into differential activity of cell signaling networks [170]. The results we show have a striking correlation with highlighting perturbation sensitive regions with the regions mutated in clinically identified oncogenic kinase mutations: the hydrophobic core in the EGFR and ErbB4 as well as the αC-β4 region in ErbB2. We have similar correlation with observed EGFR kinase mutations (L834R and del 723–729 ins S) affecting cell signaling pathways so that inhibition of the mutant kinase is a viable therapeutic strategy as well as the observed mechanisms of resistance to EGFR kinase inhibition. Furthermore, by examining the “inactive” pseudokinase ErbB3 and characterizing its weak activity, we justify an alternate means of resistance to ErbB TKI inhibition and propose that targeting of ErbB3 may represent a superior therapeutic strategy for certain ErbB-driven cancers. Therefore, multiscale modeling is a tool that can not only link kinase domain fluctuations with signaling effects to help contextualize mutations found in oncogenic cell lines, but also refine therapeutic strategies to target key perturbation sensitive regions of kinase signaling, at the molecular level as well as the cellular level.

## 4. Models and Methods

Here we provide a brief summary of the hierarchical multiscale modeling scheme we have employed for describing ErbB signaling. Sections 2.1-2.6 use a fully atomistic molecular model of the kinase domains in a solvated, equilibrated system. We model the kinase domain receptor activation characteristics of the ErbB family RTKs using molecular dynamics (MD) simulations [133-135,148] with the ErbB2 and ErbB3 kinase domains created through homology modeling [133,134]. The EGFR, ErbB2, ErbB3 and ErbB4 inactive and active monomer systems were simulated for 10 ns apiece. The EGFR active dimer and ErbB2 inactive dimer systems were also simulated for 10 ns. The EGFR inactive wildtype dimer system was simulated for 30 ns while the EGFR L834R and del 723–729 ins S mutant dimer systems were simulated for 20 ns. Principal component analysis (PCA) was performed using CARMA to find the dominant fluctuations of each domain [133-135]. There are multiple measures of protein fluctuations (B-factor, RMSF, RMSD, *etc.* [171]), we choose to use RMSD here to track the specific protein deviation at each time point. Hydrophilic and hydrophobic interaction networks were characterized for each state as well [133-135,148]. The normalized water density fluctuations were calculated using the method by the Garde group [139,140]: Vol_N_*(〈N^2^〉-〈N〉^2^)/〈N〉^2^, where Vol_N_ is the volume of interest and N is the number of water molecules in the volume of interest. The water density fluctuations are normalized by the water density fluctuations of bulk water far away from the protein.

Sections 2.7 and 2.8 use a cellular model of mass-action reactions. Signaling through EGFR is modeled by combining three published models and augmented by our own set of reactions [148]: phosphorylation and docking reactions are modeled according to [172]; the MAPK pathway reactions are modeled after [173]; Akt and PI3K activation are incorporated into the model as described in [174]. We resolve phosphorylation of the active ErbB1 dimer at tyrosine site Y1068, which, when phosphorylated, is a binding site for growth-factor-receptor bound-2 (Grb2) or Grb2-associated binding protein (GAB-1) proteins, or at tyrosine site Y1173, which, when phosphorylated, is a binding site for the Src-homology-2-containing (Shc) adaptor protein.

The ErbB3 systems model, which was based on the model by Schoeberl *et al.* [167], included reactions describing ligand-induced ErbB receptor homo- and heterodimerization, receptor internalization and degradation, constitutive dimerization, and activation of the PI3K-AKT signaling pathway [133]. The ErbB kinase inhibitor lapatinib was implemented in the model according to Schoeberl *et al.* [167]; lapatinib was assumed to inhibit activation but not dimerization or ligand binding of the EGFR and HER2 kinases.

## Figures and Tables

**Table 1.** Currently known clinically identified activating or biologically significant cytoplasmic domain mutations in RTKs classified by kinase sub-domains associated with the most lethal tumor types. * denotes a loss-of-function mutation, JM: juxtamembrane domain, PL:P-loop, αC: αC-helix, αC-β4: αC-β4 loop, HC: Hydrophobic Core, AL: A-loop, AD1: Asymmetric Dimer Interface in ErbB family, AD2: Asymmetric Dimer Interface in Ret, CT: C-terminal Tail. See also Table 2.

	**Lung**	**Colon**	**Skin**	**Breast**	**Prostate**	**Leukemia**
EGFR	PL: [31,32] αC: [31-33,49] αCβ4: [50,51] HC: [31-33,49] AD1: [49]	–	αCβ4: [52]	αC: [53]	αC: [54]	–
ErbB2	αC: [55] αCβ4: [56-58]	αCβ4: [55]	–	αCβ4: [55] αC: [59]	–	–
ErbB4	–	AL: [60]	AL: [61]	AL: [60]	–	–
PDGFRα	JM: [62] CT: [63]	–	–	–	–	AL: [64]
CSF1R/Fms	–	–	–	–	–	CT: [65,66]
Kit/SCFR	JM: [67]	–	JM: [68-71] αC: [69,71] AL: [69-71]	–	–	JM: [72,73] AL: [72-75]
Flt3/Flk2	–	–	–	–	–	JM: [76-78] AL: [79,80]
VEGFR2/KDR	CT: [62]		–	TM: [81] AL: [81]	–	–
FGFR1	AL: [63]	–	–	–	–	–
FGFR2	–	–	JM: [82]* αC: [82]* AL: [82]*	–	–	–
FGFR3	–	–	AL: [83-85]	–	–	AL: [86-88]
FGFR4	AL: [62]	–	–	–	–	–
Met	JM: [62,89]	αC: [90]	–	–	–	–
EphA1-8,10	AL: [62,63] CT: [62]	–	–	–	–	–
LTK	AL: [62] CT: [62]	–	–	–	–	–

**Table 2.** Currently known clinically identified activating or biologically significant cytoplasmic domain mutations in RTKs classified by kinase sub-domains associated with the most lethal tumor types. JM: juxtamembrane domain, PL: P-loop, αC: αC-helix, αC-β4: αC-β4 loop, HC: Hydrophobic Core, AL: A-loop, AD1: Asymmetric Dimer Interface in ErbB family, AD2: Asymmetric Dimer Interface in Ret, CT: C-terminal Tail.

	**Ovary**	**Kidney**	**Thyroid**	**Gastro Intestinal**	**Neuro Blastoma**
EGFR	–	αC: [95,96]	αC: [97] AL: [97] AD1: [98]	–	–
ErbB2	αCβ4: [99-101]	–	–	–	–
ErbB4	–	–	–	αC: [60]	–
PDGFRα	–	–	–	AL: [102-104] JM: [102-104]	–
Kit/SCFR	AL: [105,106]	–	–	JM: [107-110]	–
Met	–	–	JM [111,112]	–	–
Ret	–	–	AD2: [113-116] JM: [116,117] AL: [118]	–	–
ALK	–	–	–	–	AL: [119-122] αC: [120-122]

**Table 3.** FDA approved RTK inhibitors and antibodies currently in use.

**Name**	**Target**	**Company**	**Class**
Bevacizumab (Avastin)	VEGF	Genentech Imclone/Bristol-Meyers	Monoclonal antibody
Cetuximab (Erbitux)	EGFR	Squib	Monoclonal antibody
Panitumumab (Vectibix)	EGFR	Amgen	Monoclonal antibody
Ranibizumab (Lucentis)	VEGF	Genentech	Monoclonal antibody
Trastuzumab (Herceptin)	Erb2	Genentech	Monoclonal antibody
Pegaptanib (Macugen)	VEGF	OSI/Pfizer	RNA Aptamer
Dasatinib (Sprycel)	Src/Bcr-Abl	Bristol-Meyers Squib	Small molecule
Erlotinib (Tarceva)	EGFR	Genentech/OSI	Small molecule
Gefitinib (Iressa)	EGFR	AstraZeneca	Small molecule
Imatinib (Gleevec)	Bcr-Abl	Novartis	Small molecule
Lapatinib (Tykerb)	EGFR/Erb2	GSK	Small molecule
Nilotinib (Tasigna)	Bcr-Abl VEGFR1/2/3	Novartis	Small molecule
Pazopanib (Votrient)	PDGFR/c-kit	GlaxoSmithKline	Small molecule
Sorafenib (Nexavar)	RAF/VEGFR2/PDGFRB VEGFR2/PDGFRB	Onyx/Bayer	Small molecule
Sunitinib (Sutent)	c-kit/FLT3	Pfizer	Small molecule

**Table 4.** Persistent hydrophilic interaction network in ErbB kinase monomers; bolded entries are salt bridges while non-bolded entries are hydrogen (H-bonds). The entries are aligned so homologous interactions are in line. The conserved active interactions are boxed in brown, while the sequestering interactions are boxed in orange.

**EGFR active**	**ErbB2 active**	**ErbB4 active**	**EGFR inactive**	**ErbB2 inactive**	**ErbB3 Inactive**	**ErbB4 inactive**

aC-helix A-loop interactions						

– –	– –	– –	– –	– –	– –	**E739,R841**
				
**E734,K851**	**E766,K883**	**E739,K856**	– –	– –	– –	– –

D737,K836	D769,R868	– –	– –	– –	– –	D742,R841
E738,F832	– –	E743,F837	– –	– –	– –	– –
			
– –	– –	– –	**E738,K836**	– –	– –	**E743,R841**

A-loop C-loop interactions						

– –	– –	– –	– –	G865,V842	– –	– –
– –	– –	– –	– –	– –	– –	G838,R817
			
L834,R812	L866,R844	L839,R817	– –	– –	– –	– –

– –	– –	– –	L834,D813	– –	– –	– –
			
K836,V810	R868,V842	R841,V815	– –	– –	– –	– –

– –	– –	– –	– –	– –	**D838,R814**	– –
**E848,R812**	– –	– –	– –	– –	– –	– –
– –	– –	– –	K851,R812	– –	– –	– –

C-loop C-loop interactions						

– –	H843,D845	– –	– –	– –	H813,N815	– –
– –	– –	– –	**R812,D813**	R844,D845	– –	– –
			
**D813,R817**	D845,R849	**D818,R822**	– –	– –	– –	– –

D813,N818	– –	– –	– –	– –	N815,N820	– –
A815,N818	A847,N850	A820,N823	A815,N818	A847,N850	A817,N820	– –
– –	A848,V851	– –	– –	– –	– –	– –

aC-helix interactions

– –	A763,S760	– –	– –	– –	– –	– –
– –	E766,R756	– –	– –	– –	– –	– –
			
**E738,K721**	**E770,K753**	**E743,K726**	– –	– –	– –	– –

– –	– –	– –	M742,L753	M774,L785	– –	M747,L758
A743,L679	– –	A748,Q684	– –	– –	– –	– –
– –	– –	– –	– –	– –	– –	A748,R757

A-loop interactions						

– –	– –	D836,K726	– –	**D863,K753**	**D833,K723**	**D836,K726**
			
– –	– –	D836,T835	– –	– –	– –	– –
			
L838,R808	L870,R840	L843,R813	– –	– –	– –	– –

– –	D871,R840	– –	– –	– –	– –	– –
– –	– –	– –	– –	– –	**D844,K853**	– –
– –	– –	K848,T873	– –	– –	– –	– –
**K843,D932**	– –	K848,D937	– –	– –	– –	– –
– –	E876,R898	– –	– –	– –	– –	– –
– –	– –	E849,K871	– –	– –	– –	– –
Y845,Y867	– –	Y850,F872	– –	– –	– –	– –
– –	– –	A852,R870	– –	– –	– –	– –
– –	**D880,R897**	**D853,R870**	**E848,R865**	**D880,R897**	– –	**D853,R870**
– –	– –	– –	– –	– –	– –	G855,E730
– –	– –	– –	– –	K883,E757	– –	– –
– –	– –	– –	– –	– –	– –	**K856,E844**
